# Cardio-oncology: Shared Genetic, Metabolic, and Pharmacologic Mechanism

**DOI:** 10.1007/s11886-023-01906-6

**Published:** 2023-07-26

**Authors:** Yiqi Zhao, Hao Jia, Xiumeng Hua, Tao An, Jiangping Song

**Affiliations:** 1 Beijing Key Laboratory of Preclinical Research and Evaluation for Cardiovascular Implant Materials, Animal Experimental Centre, National Centre for Cardiovascular Disease, Department of Cardiac Surgery, Fuwai Hospital, Chinese Academy of Medical Sciences and Peking Union Medical College, Beijing, China; 2 Department of Cardiac Surgery, Fuwai Hospital, Chinese Academy of Medical Sciences and Peking Union Medical College, Beijing, China; 3 State Key Laboratory of Cardiovascular Disease, Fuwai Hospital, Chinese Academy of Medical Science, PUMC, 167 Beilishi Road, Xicheng District, 100037 Beijing, China; 4 Department of Cardiology, Fuwai Hospital, Chinese Academy of Medical Sciences and Peking Union Medical College, Beijing, China

**Keywords:** Cardio-oncology, Gene mutation, Metabolism, Cardiotoxicity

## Abstract

**Purpose of Review:**

The article aims to investigate the complex relationship between cancer and cardiovascular disease (CVD), with a focus on the effects of cancer treatment on cardiac health.

**Recent Findings:**

Advances in cancer treatment have improved long-term survival rates, but CVD has emerged as a leading cause of morbidity and mortality in cancer patients. The interplay between cancer itself, treatment methods, homeostatic changes, and lifestyle modifications contributes to this comorbidity. Recent research in the field of cardio-oncology has revealed common genetic mutations, risk factors, and metabolic features associated with the co-occurrence of cancer and CVD.

**Summary:**

This article provides a comprehensive review of the latest research in cardio-oncology, including common genetic mutations, risk factors, and metabolic features, and explores the interactions between cancer treatment and CVD drugs, proposing novel approaches for the management of cancer and CVD.

## Introduction

Cancer is the disease with the highest morbidity and mortality rate in the world [1]. The development of oncology therapies has improved long-term survival rates for cancers, such as breast cancer, lung cancer, and colorectal cancer [2]; however, identifying and intervening in the therapeutic targets and risk factors to improve long-term survival and quality of life in cancer patients remains an important research topic. Cardiovascular disease (CVD) has become the second leading cause of long-term morbidity or mortality in cancer patients [3]. The characteristics of cancer itself, treatment modalities, changes in homeostasis, and lifestyle may lead to cancer comorbidity with CVD. The cardiovascular (CV) risk in the patients’ background can be significantly increased because of the progress of cancer, especially through anticancer treatment [4]. In early-stage breast cancer, CVD is even the leading cause of death in elder women (> 66 years) with more than 5 years of survival time, followed by cancer itself [5]. Whereas in childhood cancers, survivors have 8.2 times higher later CV mortality than the same age- and sex-matched population [6]. Therefore, cardio-oncology is proposed to study the cardiotoxicity caused by anti-cancer treatment or cancer itself.

**Fig. 1:**
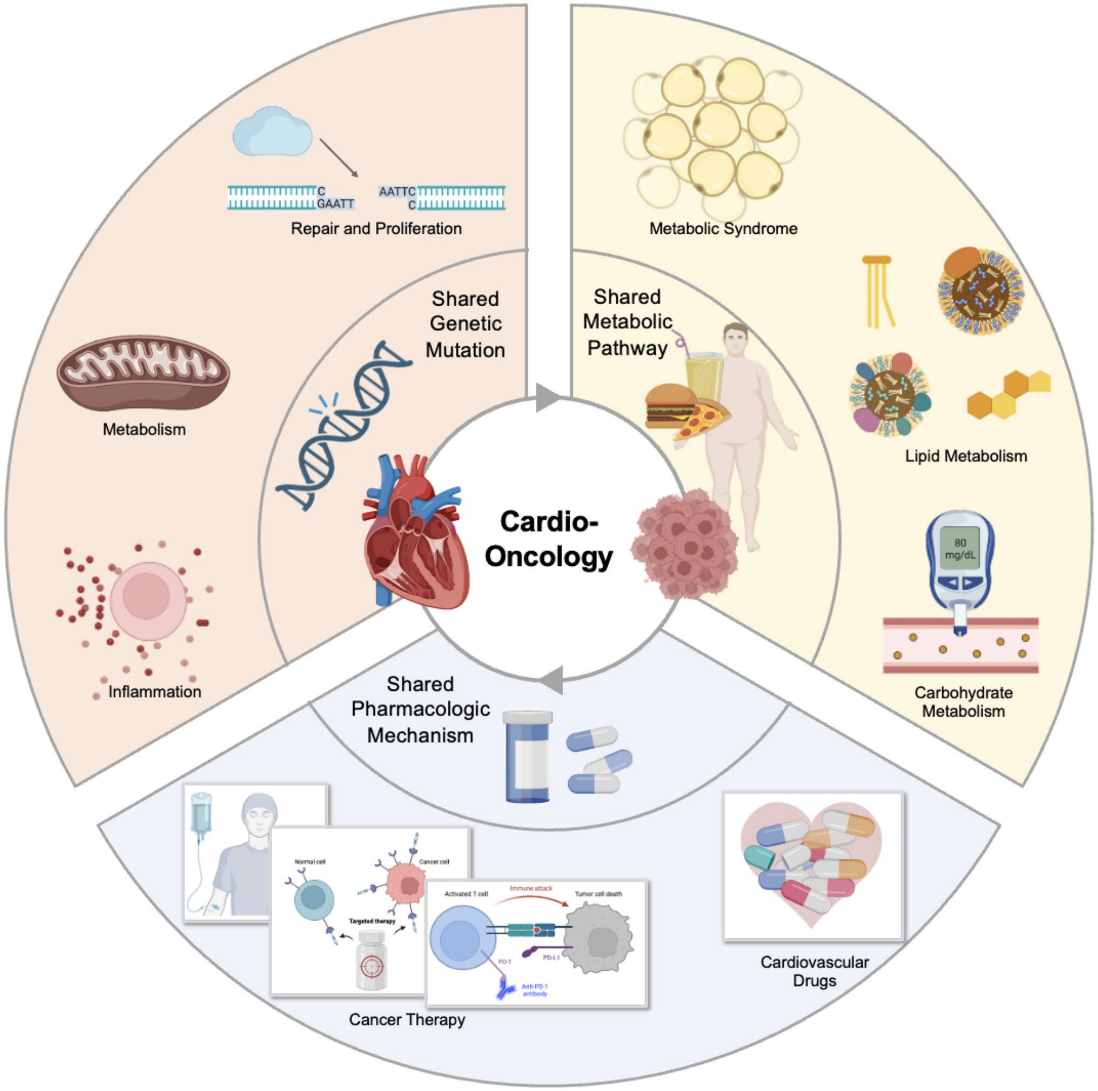
The shared mechanism of cancer-related heart disease. The research progress of cancer-related heart disease can be summarized in terms of common gene mutations, risk factors, and metabolic characteristics. The interaction between anticancer therapy and CVD drugs is also analyzed to facilitate the investigation of cardioprotection

Over the past decades, cardio-oncology research mainly focused on treatment-related cardiovascular toxicity (CVT) and the additive effects on traditional CV risk factors [7••]. It has been clarified that CVT includes heart failure (HF), reduced left ventricular ejection fraction (LVEF), diastolic dysfunction, conduction disorder, arrhythmias, and arterial and venous thrombosis, especially with newer therapies [8]. However, the interaction and connection between cancer and CVD, including the overlap of risk factors and disease pathways, are calling for more attention. Of note, the study of common metabolic substrate transformation and the gene mutation spectrum provides new insight. Moreover, the application of cardiac protection is recognized as the priority task [9]. But fundamentally, the common therapeutic targets for cancer and CVD have not yet been discovered; otherwise, two diseases could be treated at one time (Fig. 1).

## Gene Mutations

**Fig. 2:**
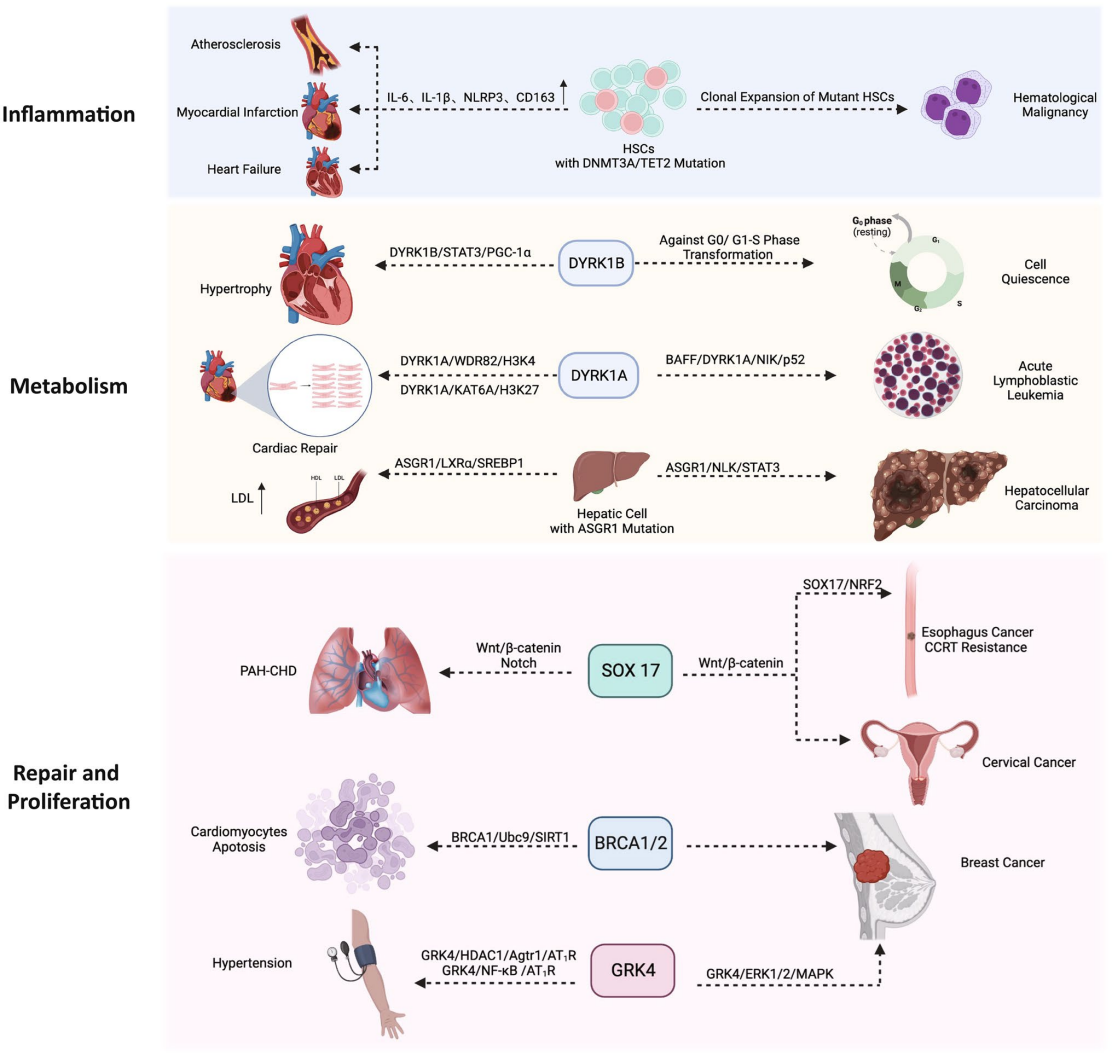
The common genetic background of cancer and cardiovascular diseases. They can be roughly summarized into inflammation-related, metabolism-related, damage-repair, and proliferation-related pathways according to the mechanism. SCLC, small cell lung carcinoma; HGSOC, high-grade serous ovarian cancer; LDL, low-density lipoprotein; PAH-CHD, pulmonary arterial hypertension in congenital heart disease

The development and progress of genomics provide an important tool for exploring the potential genetic association between cancer and CVD. In recent years, numerous studies have focused on common gene mutations in both diseases. Based on gene network analysis in a study, the most linked diseases were *JAK2*, *TTN*, and *TET2*, with the most closely associated diseases being perinatal cardiomyopathy, breast cancer, clonal hematopoiesis of indeterminate potential (CHIP), and coronary artery disease (CAD) [10]. Common gene mutations can be roughly classified into three pathways: inflammation, metabolism, and cell proliferation, all of which are closely related (Fig. 2).

### Inflammation

In tumor microenvironment (TME), complex interactions between immune cells, inflammatory cytokines, and reactive oxygen species (ROS) activate a wide range of intracellular signaling pathways [11]. Inflammation is a key pathological feature of CVDs, such as atherosclerosis (AS) [12]. Cascade reactions lead to chronic inflammation and oxidative stress, which are the common underlying mechanisms of both diseases [13]. Macroscopically, chronic systemic inflammation, as reflected by elevated C-reactive protein (CRP) levels, is a risk factor for cancer, particularly lung cancer, in patients with stable CVD [14, 15]. The complex inflammation-related genes and pathways contain many therapeutic targets. Therefore, it is essential to understand the role of common gene mutations in the two diseases.

*DNMT3A* and *TET2* mutations and abnormal expression in tumors and heart diseases are associated with inflammatory responses. These genes are the most frequently mutated in CHIP. There is a relationship between CVD and CHIP, and inflammation is one of the underlying mechanisms [16]. CHIP is characterized by the age-related acquisition and expansion of leukemia-causing mutations in hematopoietic stem cells (HSCs). The transformation rate of CHIP into hematologic malignancies is approximately 1% per year, and 10 to 15% of patients are over 65 years of age [17]. A retrospective cohort study showed that CHIP had a high incidence in patients with acute myocardial infarction (AMI) and cardiogenic shock (CS) and was associated with a poor prognosis [18]. Another study revealed that in patients with reduced LVEF and *DNMT3A* or *TET2* mutations, there is an increase in HF progression (HR = 2.79; 95%CI: 1.31–5.92) and HF-related mortality or HF hospitalization (HR = 4.41; 95%CI: 2.15–9.03; *p* < 0.001) [19].

DNMT3A is a DNA methyltransferase that epigenetically regulates hematopoiesis and serves as a tumor suppressor gene, while *TET2* encodes a tumor suppressor and transcriptional regulator that catalyzes the oxidation of 5-methylcytosine to 5-hydroxymethylcytosine in DNA [20, 21]. In protein–protein interactions, they are widely involved in chromatin organization and modification, histone modification and kinase activity regulation, and gene expression [22]. Bioinformatics analysis has indicated that CHIP-associated cpg DNA sites increase the risk of CAD by affecting lipid metabolism, immune cell recruitment, atherosclerotic plaque formation and calcification, and inflammasome production [23]. In a mouse model of chronic ischemic HF, hematopoietic or myeloid *Tet2* deficiency aggravates cardiac remodeling and function while increasing IL-1β expression, while treatment with a selective NLRP3 inflammasome inhibitor prevents the development of HF [24]. In another hematopoietic cells *Tet2*-deficient mouse model, the size of atherosclerotic plaques was significantly increased, and researchers even suggested that targeting TET2 may be a new therapeutic target for ASCVD [21].

Recent studies have also demonstrated that the expression of *DNMT3A* or *TET2* in macrophages is closely related to their response to interferon. Correspondingly, the reduction or deletion of these genes causes mitochondrial DNA damage, which activates the cGAS-STING pathway and promotes the activation of ISG in neighboring cells through IFNAR2 signaling, eventually leading to the occurrence of CVD [25]. Moreover, HSC division rates are increased in both mice and humans with AS. Elevated HSC division rates increase the risk of clonal hematopoiesis by 3.5-fold at age 70 in ASCVD patients, indicating that HSC proliferation is an important factor in the link between CVD and clonal hematopoiesis [26].

Apart from that, BRD4, a member of the BET (bromodomain and extra-terminal domain) protein family, participates in the expression of a variety of inflammatory gene pathways. It is closely associated with biological processes such as transcription regulation and chromatin remodeling [27]. By binding to acetylated histones, BRD4 induces the production of inflammatory factors such as tumor necrosis factor (TNF) and IL-6. BET proteins play vital roles in mammalian development and BET protein inhibitors have emerged as important anticancer agents. *BRD4* links to high expression of oncogenes like *MYC*, *NOTCH3*, and *NRG1* in BRD4-amplified high-grade serous ovarian cancer (HGSOC) patients [28]. BRD4-driven oncogenes promote cancer cell proliferation, genetic instability, epithelial-mesenchymal transition (EMT), metastasis, and chemotherapy resistance [29]. In small-cell lung cancer (SCLC) patients, the BRD4/ASXL3/BAP1 epigenetic axis regulates the oncogenic function of chromatin active enhancers in the SCLC-A subtype and is viewed as a novel therapeutic target [30]. In the heart, *BRD4* acts as a central regulator of the profibrotic cardiac fibroblast phenotype, establishing a p38-dependent signaling pathway for epigenetic reprogramming in HF. Inhibition of BRD4 by the selective bromodomain inhibitor JQ1 also attenuated myocardial hypertrophy and HF caused by thoracic aortic coarctation [31]. Apabetalone (RVX-208), an epigenetic modulator that targets BET proteins, is also available for treatment [32].

Inflammation’s role in cancer and CVD has long been studied. Traditional inflammation-related genes, as well as *IL-1B*, *IL-6*, *IL-8*, and *TNF-α*, are involved in the pathogenesis of both diseases [33–35]. As the understanding of the disease deepens, more and more novel genes, including *DNMT3A*, *TET2*, *BRD4*, etc., are being discovered and are expected to be the therapeutic targets for comorbidities.

### Metabolism

The current understanding of the genetic basis of cancer and CVD has focused on how metabolic dysregulation of cancer cells can extend beyond the TME and lead to systemic and cardiac-specific consequences. For example, cancer cells with *IDH1* or *IDH2* variants can produce and release increased levels of D-2-hydroxyglutarate dehydrogenase (D2-HG), while inhibition of α-ketoglutarate dehydrogenase can impair oxidative metabolism, leading to a reduction in adenosine triphosphate (ATP) supply and cardiac contractile function [36]. However, less attention has been paid to the direct effect of gene mutations on cancer and CVDs.

Mammalian dual-specificity tyrosine phosphorylation-regulated kinase (DYRK) is a subfamily of mitogen-activated protein kinase-related protein kinases (MAPKs) that has two isoforms, DYRK1A and DYRK1B [37]. *DYRK1B* gene regulates the expression of the mitochondrial electron transport chain complex, oxidative phosphorylation, energy production, and mediates cancer cell proliferation and chemoresistance [38]. *DYRK1B* is overexpressed in cancer, maintaining cell quiescence by resisting the G0/G1-S phase transition and mediating the upregulation of antioxidant gene expression to improve cell survival [38]. Moreover, *DYRK1B* plays a critical role in HF. Studies have demonstrated that *DYRK1B* is significantly upregulated in human failing myocardium and in hypertrophic mouse hearts [39]. Mechanistically, DYRK1B positively correlates with impaired mitochondrial bioenergetics by directly binding to signal transducer and activator of transcription 3 (STAT3), increasing its phosphorylation and nuclear accumulation, ultimately leading to the downregulation of PGC-1α. Furthermore, inhibition of DYRK1B or STAT3 activity can restore cardiac function by restoring mitochondrial bioenergetics.

*DYRK1A* has been found to interact with TNF receptor-associated factor 3 (TRAF3) and mediate its phosphorylation, leading to the activation of the non-canonical nuclear factor kappa B (NF-κb) signaling pathway [40]. This, in turn, affects the development and occurrence of acute lymphoblastic leukemia (ALL) and offers a new concept and target for the treatment of the disease. And in the myocardial infarction (MI) mouse model, knocking down *DYRK1A* has been found to lead to cell cycle activation in cardiomyocytes and enhanced expression of many genes controlling cell proliferation [41]. This, along with increased H3K4me3 and H3K27ac deposition in the promoter regions of these genes, highlights the crucial role of *DYRK1A* in cardiac repair and suggests that it may serve as a therapeutic target for cardiomyopathy [41].

*ASGR1*, a tumor suppressor gene regulated by DNA methylation, is downregulated in hepatocellular carcinoma (HCC) and correlated with tumor size, grade, and survival time [42]. By promoting the binding of NLK to STAT3 and inhibiting the phosphorylation of STAT3, ASGR1 inhibits the progression of HCC both in vitro and in vivo. Interestingly, *ASGR1* also functions in lipid metabolism [43]. Expressed only in hepatocytes, ASGR1-deficient mutations are associated with lower cholesterol and reduced risk of CVD [44]. This is achieved by stabilizing LXRα (liver X receptors), lowering serum and hepatic lipid levels, and blocking glycoprotein endocytosis and lysosomal degradation. Additionally, it reduces the level of amino acids in lysosomes, inhibits mammalian target of rapamycin (mTOR), and activates AMP-activated protein kinase (AMPK).

Finally, metabolic reprogramming, a hallmark of cancer and HF, is critical in optimizing nutrient utilization to meet the energy demands for proliferation, biosynthesis, and energy supply. As risk factors for cancer and CVD overlap significantly, future studies can analyze disease pathways more deeply [45].

### DNA Damage Response and Cell Proliferation

DNA damage is a common feature in a range of CVD and cancer, eliciting cellular responses such as detection of damage, cell cycle arrest, DNA repair, cell senescence, and apoptosis [46•, 47]. While transient DNA damage response (DDR) from transient DNA damage is beneficial, continuously activated DDR promotes the onset and progression of the disease.

The WNT/β-catenin signaling pathway involved in DNA damage and cell proliferation is crucial in cancer and CVD, controlling gene expression in development, stem cell homeostasis, and diseases such as cancer [48]. The downstream molecule SRY-related HMG box (SOX) transcription factor is an environment-dependent regulator of WNT-reactive transcription in early cell fate decisions. *SOX17* can inhibit the proliferation and tumorigenesis of cervical cancer by downregulating the activity of the WNT/β-catenin signaling pathway through β-catenin trans-inhibition [49]. In esophagus squamous cell carcinoma (ESCC), a novel SOX17low/NRF2high signature was identified, where SOX17 acts as a transcriptional repressor of NRF2, presenting a promising strategy for targeting the SOX17/NRF2 axis to overcome drug resistance [50]. The transcription factor encoded by *SOX17* is also involved in the Notch signaling pathway during development and is a new risk gene for pulmonary arterial hypertension in congenital disease (PAH-CHD) and idiopathic/familial PAH [51].

Germline *BRCA1/2* mutations associated with hereditary breast and ovarian cancers also associate with CVD [52]. Women with *BRCA* mutations had a significantly increased risk of CVD compared with women without *BRCA* mutations who did not undergo bilateral oophorectomy (BO) (HR = 1.82, 95%CI: 1.18–2.79) [53]. The *BRCA1/2* gene regulates the repair of DNA damage, which occurs in all types of cells, including vascular endothelial cells and cancer cells [54]. At present, some studies have combined *BRCA* mutations with the susceptibility to myocardial injury after radiotherapy and chemotherapy, but the specific mechanism needs to be further investigated.

*GRK4* is associated with both breast cancer and hypertension. In hypertension, G-protein-coupled receptor (GPCR)-mediated regulation of kidney and arterial function affects blood pressure [55]. High expression of *GRK4* in breast cancer cells may activate the β-arrestins-mediated MAPK signaling pathway, suggesting that *GRK4* may be involved in the development of breast cancer [56]. In addition, it is found that low expression of *GRK4* in liver cancer tissues correlates with poor prognosis, and *GRK4* overexpression inhibits the proliferation and migration of liver cancer cells [57].

The vast gene regulatory network has long sought to identify common regulatory targets to achieve a “multiple benefits with one hit” therapeutic effect. Genetic testing allows cancer patients to receive more targeted treatment and offers the possibility of early warning and intervention for potential CVD.

## Metabolic Pathway

**Fig. 3:**
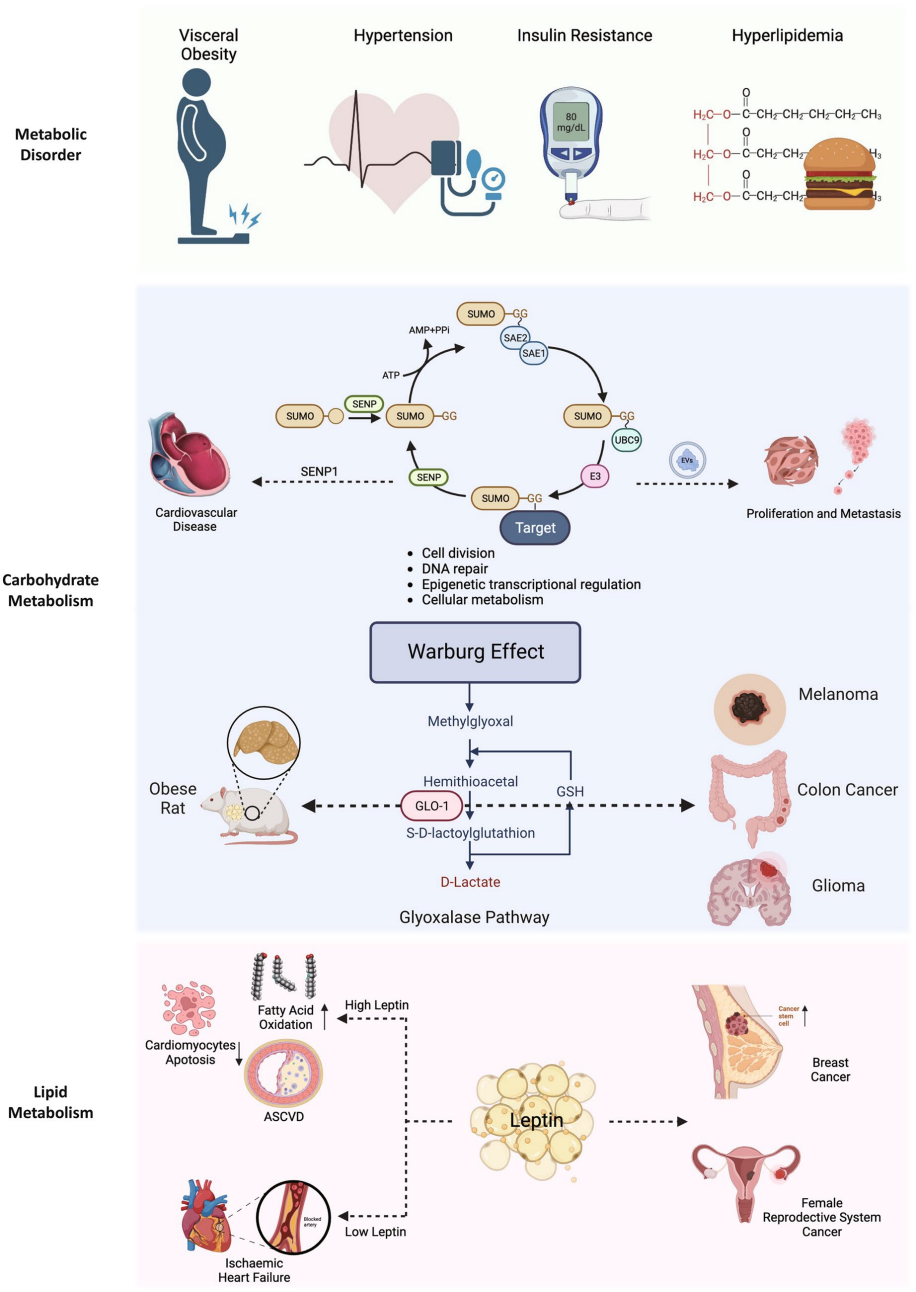
The commonality of metabolic pathways in cancer and cardiovascular disease. The transformation of metabolic patterns, specifically carbohydrate and lipid metabolism, is a common feature in cancer and CVD. Anti-cancer treatment may also lead to further metabolic disorders

The transformation of metabolic patterns, specifically carbohydrate and lipid metabolism, is a common feature in cancer and CVD [58]. However, anti-cancer treatment may also lead to further metabolic disorders. Currently, research on cancer cells and CVD focuses on the relationship between obesity, hyperlipidemia, diabetes, and the occurrence and development of cancer. The molecular mechanisms of metabolic reprogramming involving glucose, fatty acids, ketone bodies, amino acids, and other substrates are being investigated (Fig. 3).

### Metabolic Disorders

Metabolic disorders, particularly metabolic syndrome, represent a group of common metabolic abnormalities and disease states such as hypertension, hyperglycemia, obesity, and dyslipidemia [59]. It relates to the onset and progression of multiple chronic diseases, including cancer and CVD.

Obesity directly contributes to incident cardiovascular risk factors, including dyslipidemia, type 2 diabetes, hypertension, and sleep disorders [60]. In addition, adipose tissue secretes a variety of adipokines that affect the behavior of immune cells and tumor progression, setting the relationship between obesity and cancer [61]. However, some studies have shown that there is also an “obesity paradox” in cancer. High body mass index (BMI) does not increase the risk of progression or death in patients with early colorectal cancer [62], and may even be a protective factor [63]. This finding is consistent with the observation in patients with HF [64]. Higher BMI is associated with improved outcomes in patients with HF despite being a risk factor for the onset of HF. The possible reason is that obesity may become a metabolic reserve in cancer patients, resisting body consumption and the side effects of cachexia.

Hyperglycemia is a well-recognized risk factor for CVD, with a strong clinical relevance, and is associated with both CVD morbidity and mortality [65, 66]. The damage of endothelial, vascular smooth muscle and myocardial function caused by diabetic hyperglycemia and oxidative stress accelerates the occurrence of CVD [67]. Hyperglycemia can increase blood viscosity, aggravating the risk of thrombosis and myocardial ischemia [68]. And it is now well established that diabetes is associated with colorectal, breast, endometrial, liver, pancreatic, and bladder cancers [69]. However, the exact pathogenesis of diabetes-mediated CVD and cancer is still unclear. It has been suggested that the mechanism involves the promotion of cell proliferation by insulin-like growth factor 1 (IGF-1). IGF-1 also stimulates smooth muscle cell migration and proliferation, which is part of the pathogenesis of AS [70].

### Carbohydrate Metabolism

#### Glucose Metabolism Reprogramming

Glucose metabolism reprogramming is reflected in the “Warburg effect” in malignant tumors, in which the pattern of cellular metabolism shifts from oxidative phosphorylation to glycolysis during nutrient deprivation [71]. In HF, early metabolism is characterized by enhanced glucose utilization and decreased fatty acid oxidation (FAO), leading to increased lactate secretion. The dependence on the ketone bodies of the heart will increase, with a reduced ability to use fatty acids [72]. However, ketone bodies are also cancer metabolites, which provide “fuel” for cancer metabolism. Ketone inhibitors have been considered new therapeutic targets, and *HMGCS2*, *ACAT1/2*, and *OXCT1/2* are metabolic oncogenes [73].

Another important consequence of the “Warburg effect” is the intracellular formation of methylglyoxal (MGO), a precursor of advanced glycation end products (AGE) [74]. Cancer cell with a high glycolytic rate requires a high detoxification rate of MGO. Glyoxalase 1 encoded by the *Glo1* gene acts as a rate-limiting enzyme [75]. High *Glol* expression may be a mechanism for the survival and growth of tumors with high rates of glycolysis and associated high flux of MGO formation [76]. *Glo1* can affect glucose and lipid metabolism in spontaneously hypertensive rats (SHR) [77]. Compared with SHR wild-type rats, ShR-glo1 + / − rats had a lower epididymal fat weight and reduced ectopic fat accumulation in the liver and heart. *Glo1* is also recognized as an oncogene. Its high expression links to the progression of colorectal cancer and melanoma [78, 79]. Besides, studies on glioma (GBM) have shown that *Glo1* inhibition may be a potential molecular target to improve the efficacy of anticancer therapy [80].

#### SUMOylation: a New Player in Disease Pathology

Signal transduction, especially the kinase-related pathway, has an important role in promoting diabetes and CVD pathology [81]. SUMOylation is a hotspot in recent years. Small ubiquitin-related modifier (SUMO) is a 10-kDa post-translational modification protein [82]. Modification of the target protein can lead to conformational changes, thereby regulating protein function. Kinases such as protein kinase C (PKC), extracellular signal-regulated kinase 5 (ERK5), and 90 kDa ribosomal s6 kinases (p90RSK) are activated under diabetic conditions, being continuously modified by SUMOylation, and play a central role in regulating the signal transduction network [83]. SUMOylation also modifies insulin gene expression, glucose metabolism, and insulin exocytosis in islet β cells under physiological conditions [84].

Diabetes accelerates vascular dysfunction, leading to cardiomyopathy. Studies have found that SUMO is enhanced and X-box binding protein 1 (XBP1) nuclear translocation is impaired in a diabetic environment. Besides, Zhou et al. showed that loss of the endothelial-specific SUMO endopeptidase sentrin-specific protease 1 (SENP1) reduced pathological angiogenesis and tissue repair during hindlimb ischemia [85]. In the diabetic environment, SENP1 is downregulated, while vascular endothelial growth factor receptor 2 (VEGFR2) is hyper-SUMOylated. Expression of the non-SUMOylated form of VEGFR2 rescued the angiogenic defect in diabetic mice [85].

SUMO is also widely explored in cancers [86]. SUMOylation is elevated in pancreatic ductal adenocarcinoma (PDAC) patient samples and is a reversible post-translational modification required for cell cycle progression [87]. Pharmacological inhibition of the SUMO pathway is a potential strategy to target PDAC by inhibiting cancer cell cycle progression and activating anti-cancer immunity by inducing interferon signaling [87]. In cancer metastasis, SUMOylation is an inducer that sorts bioactive molecules into extracellular vesicles (EVs), triggers lymphangiogenesis, and then drives tumor metastasis via lymph node (LN) [88]. The EV-mediated ELNAT1/UBC9/SOX18 regulatory axis promotes lymphangiogenesis and lymph node metastasis in bladder cancer in a SUMOylation-dependent manner [89].

### Lipid Metabolism

#### Effects of Dyslipidemia on CVD and Cancer

The direct impact of hyperlipidemia on ASCVD has been well established [90]. For cancer, studies have reported that hypercholesterolemia affects the pathogenesis of cancer by selecting cells resistant to ferroptosis [91]. Among patients with endometrial and ovarian cancer receiving endocrine therapy, there is an increased risk of CVD, of which dyslipidemia is the most common [80]. Among patients with testicular cancer, cisplatin-based radiotherapy or chemotherapy conferred an increased long-term risk for AS (HR = 4.8; 95% CI, 1.6–14.4) [92]. 27-Hydroxycholesterol (27-HC), a major metabolite of cholesterol that is also an estrogen receptor (ER) and LXR ligand, increases the incidence of ER-dependent growth and LXR-dependent metastatic breast cancer in mouse models [93]. Research on the impact of hyperglycemia on cancer and CVD is warranted.

#### Effects of Leptin and Adiponectin

Adipokines, especially those related to leptin and adiponectin metabolic pathways, play important roles in the process of regulation. Leptin is a 16kd adipokine produced by the obesity (*OB*) gene and is mainly secreted by adipose tissue [94]. Leptin at physiological concentrations can have a variety of beneficial effects to maintain glucose stability, such as binding to leptin receptor (LepR) in the hypothalamic region of the brain, acting as a neurotransmitter to convey energy status and suppressing appetite, and promoting energy expenditure [95]. Leptin is also secreted by cardiomyocytes [96], and there are leptin receptors in cardiomyocytes [97], suggesting that leptin directly regulates the metabolism of the heart to maintain normal cardiac function. Mouse models lacking cardiomyocyte leptin and its receptor result in severe CVD [98]. However, chronic elevated pathological levels or hyperleptinemia are risk factors for the development of CVD [99]. The level of circulating leptin is directly proportional to the amount of adipose tissue and is one of the indicators for the evaluation of obesity [100]. Endothelial leptin signaling is involved in cardiac fibrosis and functional deterioration by inhibiting endothelial cell autophagy and promoting endothelial dysfunction [101].

The effect of leptin has been increasingly mentioned in women’s cancer [102]. Leptin-mediated signaling through JAK/STAT3 and PI3K/AKT/mTOR pathways can increase the frequency of cancer stem cell (CSC), chemotherapy resistance, and tumor progression in breast cancer [103]. In obese mice with spontaneous breast cancer, leptin and PD-1 inhibit the glycolysis of CD8 + T cells via STAT3-driven increased FAO [104]. In HCC, berberine(BB) may play a preventive role in hepatocellular carcinoma by antagonizing the ATX-LPA-LPAR2-p38-leptin axis in the liver [105].

## Cardiovascular Diseases Caused by Anti-cancer Drugs

**Fig. 4:**
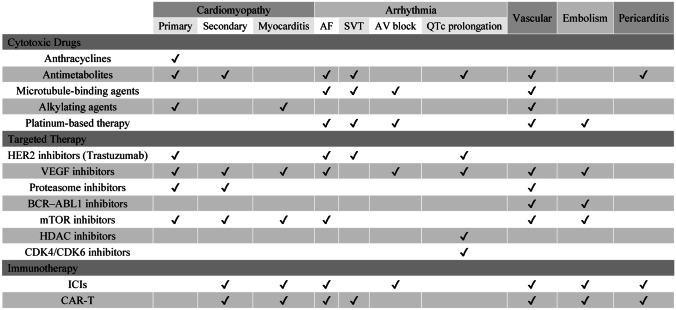
An overview of cardiovascular toxic effects associated with cancer therapies. Many cancer therapies are associated with adverse effects and complications throughout the cardiovascular system, with some having very limited cardiovascular toxicity and others having very broad cardiovascular toxicity. HER2, human epidermal growth factor receptor 2; VEGF, vascular endothelial growth factor; mTOR, mechanistic target of rapamycin; HDAC, histone deacetylase; CDK, cyclin-dependent kinases; ICI, immune checkpoint inhibitor; CAR-T, chimeric antigen receptor T-cell therapy; AF, atrial fibrillation; SVT, supraventricular tachycardia; AV, atrioventricular; QTc, corrected QT interval

Previous studies have explored the cardiotoxicity of anti-cancer treatments including mechanisms, biomarkers, and cardiac protection therapy. The emergence of novel drugs also brings new problems in cardiac protection. The types of diseases can be divided into cardiomyopathy, arrhythmia, vascular diseases, hypertension, valvular diseases, thrombotic disease, and others [106]. Here, we mainly focus on cardiomyopathy to find the commonality in different drugs and provide a systematic reference for cardioprotective drugs (Fig. 4).

### Cardiomyopathy

Cancer therapy-induced cardiomyopathy can be described as primary cardiomyopathy (direct damage to myocardial cells), secondary cardiomyopathy (perfusion changes, innervation, hormonal regulation), and myocarditis (inflammatory cell infiltration) [107]. In addition, there are still specific types of cardiomyopathies, such as cardiac amyloidosis (CA), a common injury caused by proteasome inhibitors, which has been classified as the most serious cardiotoxicity in the guidelines and needs to be closely monitored [7••].

The traditional chemotherapy drugs represented by anthracyclines are mainly primary cardiomyopathy [107]. It (1) inhibits DNA replication and RNA synthesis by intercalating between the bases of DNA double-strand; (2) inhibits topoisomerase II and hinders DNA replication and transcription; (3) produces free radicals after chelating iron ions to destroy DNA, protein, and cell membrane structure [108]. The mechanisms of anthracycline-related cardiotoxicity overlap with their anticancer effects to some extent. But studies have shown the prominent role of myocardial mitochondrial damage in impaired cardiac function [109], as ROS is the key mediator of chemotherapy-induced cardiotoxicity. Mitochondria are abundant in cardiomyocytes and are the main source of ROS [110]. At the same time, the transformation of damaged mitochondria through autophagy is essential for maintaining the structure and function of cardiomyocytes [111]. In addition, a decrease in the Bcl-2/Bax ratio, which leads to mitochondrial pore formation and activation of apoptotic pathways, also plays a role in cardiac injury [112].

Left ventricular (LV) dysfunction and HF are the common symptoms of anthracyclines-induced cardiotoxicity (AIC), most of which occur within 1 year of treatment and are considered early-onset chronic cardiotoxicity [113]. In recent years, the study of genetic susceptibility to cardiotoxicity is an emerging hotspot [114]. For example, alterations in gene expression (*CYBA*, *GSTA1*, *NCF4*, *RAC2*, *ABCC1*, *ABCC2*, and *CAT*) lead to mitochondrial dysfunction, resulting in increased ROS production and ultimately myocyte injury [115]. Pharmacogenomic testing has been recommended for all pediatric cancer patients with indications for treatment with DOX [116].

Cardiotoxicity caused by targeted therapies has a unique clinical phenotype. Trastuzumab, a monoclonal antibody against human epidermal growth factor receptor 2 (HER2), plays an important role in the treatment of metastatic breast cancer [117]. Trastuzumab can cause a decrease in LVEF due to the expression of ErbB2/ErbB4 receptors in cardiomyocytes [118]. NRG-1 has a protective effect on myocardial stress, but the pathway can be disrupted by HER2 antibody, leading to cardiotoxicity [119]. It is independent of the cumulative dose of trastuzumab and is generally reversible. Anthracyclines in combination with trastuzumab have shown a higher incidence of cardiotoxicity [120], possibly because of the disruption of the NRG-1 pathway. In the trial conducted by Slamon et al., concomitant administration of trastuzumab with anthracyclines provided significant clinical benefit in patients with advanced breast cancer but resulted in a high incidence of HF (28%) [121]. Clinically dose of trastuzumab significantly impaired the contractile and calcium-processing properties of induced pluripotent stem cell cardiomyocytes (iPSC-CMs) without inducing cardiomyocyte death or sarcomeric deranging [122]. RNA sequencing and functional analysis revealed that mitochondrial dysfunction and altered cardiac energy metabolism pathways were the primary causes of the trastuzumab-induced cardiotoxic phenotype.

Vascular endothelial growth factor inhibitors (VEGFi), such as bevacizumab, have been reported to cause reduced ventricular function, and even Takotsubo syndrome, which could be ascribed to its modulation of nitric oxide and catecholamine [123]. While VEGF-TKI (tyrosine kinase inhibitors) is considered to be the most closely related to cardiomyopathy among TKIs, studies in human induced pluripotent stem cell cardiomyocytes (hiPSC-CMs) suggest that insulin receptor signaling can act as a compensatory pathway in the treatment of VEGF signaling inhibition [124].

## Effects of Cardiovascular Drugs on Cancers

Guideline-based HF therapy, including angiotensin-converting enzyme inhibitors/angiotensin receptor blockers (ACEI/ARB), β-receptor blocker (βRB), sodium-glucose cotransporter-2 (SGLT2) inhibitors, etc., is recommended for patients with cancer therapy-related cardiac dysfunction (CTRCD) who develop symptoms during anthracycline-based chemotherapy [7••]. While studying the cardiovascular protective effects of drugs, such drugs may also have potential effects on the tumor itself.

## ACEI/ARB

ACEI/ARB/βRB has a significant cardioprotective effect in breast cancer patients with HER2-targeted therapy with reduced LVEF [125]. In a clinical trial, in 20 patients receiving HER2-targeted therapy and cardiac protection at the same time, LVEF increased gradually from an average of 49% at enrolling to 55% at 12 months (*p* < 0.001) [126]. However, 10% of patients may develop moderate-to-severe HF. Furthermore, different types of ACEI/ARB may bring different clinical effects. Perindopril and bisoprolol have been proven to prevent the decline in LVEF related to cancer therapy in patients with HER2-positive early breast cancer treated with trastuzumab [127]. Yet, these pharmacologic treatments did not prevent trastuzumab-mediated left ventricular remodeling.

In addition to their cardioprotective effect, ACEI drugs may also have a role in maintaining vascular function, allowing adequate delivery of chemotherapy drugs to tumor cells. In a study to explore the protective effect of zofenoprilat on DOX-induced coronary microvascular endothelial injury, endothelial cells in the exposure of DOX (0.1–1 μm) showed impaired cell survival. ERK1/2-related p53 activation, but not ROS, is responsible for DOX-induced caspase-3 cleavage. Impaired p53-mediated apoptosis was reversed by zofenoprilat treatment. In addition, the treatment of endothelium-tumor coculture with zofenoprilat did not affect the antitumor efficacy of DOX given the tumor environment. ACEI zofenoprilat has a protective effect against DOX-induced endothelial injury without affecting its antitumor efficacy.

## MRA

The protective effects of mineralocorticoid receptor antagonists (MRA) or ACEI on acute and chronic doxorubicin-induced cardiotoxicity (DIC) in mice have been reported in animal models [128]. In both DIC models, DOX-induced myocardial atrophy, reduced left ventricular volume, reduced cardiomyocyte volume, and cardiac insufficiency. In the acute model, MRA and ACEI were not protective against these manifestations of DIC. In the chronic model, enalapril treatment protected against cardiac dysfunction and cardiomyocyte atrophy, and was associated with PI3K/AKT/mTOR pathway activation and normal levels of connective tissue growth factor (CTGF). MRA eplerenone was also reported to prevent left ventricular dysfunction [129]. RNA-sequencing data extracted from isolated cardiomyocytes revealed DOX’s inhibitory effect on gene expression, which was prevented by MR deletion. MRA may be suitable for the prevention of DIC. Further clinical trials may be applied for it.

### βRB

Unlike ACEI, βRB may also have anti-cancer effects while protecting the heart [130]. It has been found that the sympathetic nervous system drives breast cancer progression through β-adrenergic receptor signaling pathways [131]. The effects of βRB carvedilol were explored in a mouse model of breast cancer and a large cohort of breast cancer patients (*n* = 4014). Carvedilol reduces primary tumor growth and metastasis in mouse models of breast cancer and prevents invasion of breast cancer cell lines through the blockage of sympathetic nervous system activation. The retrospective analysis found that women who used carvedilol at the time of diagnosis of breast cancer (*n* = 136) had a reduction in breast cancer-specific mortality (*p* = 0.076) compared with those who did not use carvedilol (*n* = 3878), providing a theoretical basis for exploring the use of the carvedilol as a new strategy to slow cancer progression. Another prospective, randomized, double-blind, placebo-controlled study was designed to evaluate the role of carvedilol in the prevention of DIC [132]. A total of 200 patients were enrolled, and the incidence of cardiotoxicity was 13.5–14.5%. There was no significant difference in the changes of LVEF and brain natriuretic peptide (BNP) between the two groups, the level of troponin I (TnI) in the carvedilol group was lower (*p* = 0.003), and the incidence of diastolic dysfunction in the carvedilol group was lower (*p* = 0.039).β-adrenergic receptors activate cAMP/PKA/MAPK pathways in pancreatic cancer cells, suggesting that β-receptors may play a role in cancer invasion, while β-blockers may inhibit invasion and proliferation [133]. β2 receptor antagonists significantly alter the expression of VEGF, cyclooxygenase 2 (COX-2), matrix metalloproteinase-2 (MMP-2), and MMP-9, thereby inhibiting invasion and proliferation, which may be a novel strategy for the prevention and treatment of cancer. And carvedilol has also been shown to have a protective effect against skin cancer based on promoting β-suppressor protein-mediated processes such as ERK phosphorylation [134]. Research showed that carvedilol and alprolol blocked EGF-induced phosphorylation and activation of c-Jun/AP-1 and ELK-1. Both carvedilol and alprolol effectively inhibited EGF-induced tumor transformation of JB6 P + cells.

### SGLT2 Inhibitors

SGLT2 inhibitors, specifically their relevance to metabolic reprogramming, play a vital role in cardiorenal protection [135]. SGLT2 inhibitors improve outcomes in patients with HF and are protective in patients undergoing anti-cancer treatments. In a study of 3033 patients with diabetes mellitus (DM) and cancer who received anthracyclines, the group treated with SGLT2 inhibitors had a lower incidence of cardiac events than the control group (*p* = 0.025) and also lower overall mortality (*p* < 0.001), showing preliminary evidence that SGLT2 inhibitors are associated with a lower rate of cardiac events and a moderate safety profile [136]. In another study, empagliflozin (EMPA), an SGLT-2 inhibitor, improves myocardial strain and reduces myocardial fibrosis and pro-inflammatory cytokines in DOX-treated non-diabetic mice [137]. Mechanically, it improves cardiac function by participating in NLRP3 and MyD88-related pathways. EMPA also showed a protective effect against sunitinib-induced cardiac dysfunction in mice via the AMPK-mTOR signaling pathway [138]. However, these effects still need to be validated in clinical trials.

Recent evidence suggests that tumor cells also express sodium-glucose transporters in addition to classical glucose transporters [139]. Although high glucose uptake and glycolysis rates are common in tumors, the pharmacology of blocking glucose utilization in tumor therapy is not well defined. SGLT inhibitors have the potential to exert antitumor activity by restricting glucose uptake. Canagliflozin, another SGLT-2 inhibitor, can inhibit the proliferation of breast cancer cells, and its anti-proliferation effect was not affected by glucose availability and SGLT2 expression level [140]. Canagliflozin reduces oxygen consumption and glutamine metabolism through the citric acid cycle. The antiproliferative effect of canagliflozin is related to the inhibition of respiration promoting glutamine metabolism, which is an unreported mechanism for the potential antitumor effect.

### Emerging Drugs and Treatments

Phosphodiesterase 5 (PDE5) inhibitors have a strong protective effect against myocardial ischemia/reperfusion injury, DIC, and improve the efficacy of stem cells in myocardial repair [141]. Meanwhile, PDE5 is highly expressed in a variety of human cancers and is thought to be involved in tumor progression [142]. Recent studies suggest that PDE5 inhibitors, as an important adjunctive drug, enhance the sensitivity of certain types of cancer to chemotherapy  drugs or inhibit adverse drug reactions [143, 144]. Many clinical trials of PDE5 inhibitors have focused on potential cardiovascular and anti-cancer benefits [145]. Interestingly, it can also reduce the side effect of some anti-cancer drugs. Through high-throughput screening, PDE5 inhibitors, including sildenafil, were found to be non-toxic and effective inhibitors of romidepsin-induced EBV reactivation, with downstream cGMP/PKG pathways negatively associated with EBV reactivation in NKTL cells [146].

Calpain is a family of calcium-dependent mercaptoproteases associated with CVD [147]. Proapoptotic effects of inhibiting calpain are associated with decreased protein kinase B (AKT) protein and mRNA expression and reduced phosphorylation of glycogen synthetase kinase-3β (GSK-3β) in DOX-treated cardiomyocytes [148]. Myocardial dysfunction in calpains knock-out mice was aggravated by overexpression of calpain inhibitors, and the 5-day mortality rate in transgenic mice (29.16%) was higher than in wild-type litter mice (8%). Overexpression of calpastatin may enhance DOX-induced myocardial injury by inhibiting calpain.

Last but not least, pharmacogenomics, as an individualized approach to determining genetic differences in drug configuration and treatment response, can identify markers that predict adverse effects, enhance drug efficacy, reduce toxicity, and analyze genetic susceptibility [149]. In the presence of vincristine treatment, children who express CYP3A5 have been shown to have a significantly lower risk of peripheral neuropathy than those who express the inactive form or CYP3A4 isoform, suggesting that pretreatment genetic testing is a reliable means of risk stratification [150].

## Conclusion

In summary, we review the research progress of cancer-related CVD from the aspects of common gene mutations, risk factors, and metabolic characteristics. We also analyzed the interactions between anticancer treatment and CVD drugs and proposed the benefits of novel cancer and CVD treatment. Future research directions include risk stratification of cancer patients, identifying specific patterns of early myocardial injury, serum markers, and imaging. It can also be the improvements in interventional diagnostic methods and the application of artificial intelligence (AI) analysis to identify individuals at risk of cancer and new parameters to predict risk and response to specific cardioprotective interventions.

## Data Availability

Not applicable.

## Abbreviation

AIC: Anthracyclines-induced cardiotoxicity
CAR: Chimeric antigen receptor
CHIP: Clonal hematopoiesis of indeterminate potential
CRS: Cytokine release syndrome
CTRCD: Cancer therapy-related cardiac dysfunction
CVD: Cardiovascular disease
DIC: Doxorubicin-induced cardiotoxicity
FAO: Fatty acid oxidation
HF: Heart failure
LVEF: Left ventricular ejection fraction
